# Posterolateral Rotatory Instability of the Elbow: A Practice-Focused Narrative Review

**DOI:** 10.7759/cureus.96151

**Published:** 2025-11-05

**Authors:** Ahmad A Quzli, Ahmad M Elheet, Rawand A Quzali

**Affiliations:** 1 Trauma and Orthopaedics, Wirral University Teaching Hospital NHS Foundation Trust, Wirral, GBR; 2 Accident and Emergency, Blackpool Victoria Hospital NHS Foundation Trust, Blackpool, GBR; 3 Physiotherapy, Abu Dhabi Health Services Company (SEHA), Abu Dhabi, ARE

**Keywords:** cubitus varus deformity, elbow biomechanics, elbow instability, lateral ulnar collateral ligament, posterolateral rotatory instability

## Abstract

Posterolateral rotatory instability (PLRI) is the most common pattern of elbow instability and an underdiagnosed cause of persistent lateral elbow pain. Under-recognition is common because radiographs may be normal, and the instability is dynamic. This narrative review synthesises contemporary evidence on the lateral collateral ligament (LCL) complex, with emphasis on the lateral ulnar collateral ligament (LUCL), and integrates applied anatomy, pathomechanics, clinical presentation, examination, imaging choices, and management. Diagnosis is primarily clinical, led by history and provocative testing and supported by targeted static imaging (plain radiographs, computed tomography (CT), magnetic resonance imaging (MRI)) and by dynamic modalities (fluoroscopy or ultrasound) when static studies are equivocal. Conservative non-operative care comprises activity modification, targeted physiotherapy, and time-limited bracing for low-grade, low-demand, and improving cases. Symptomatic or chronic instability is treated with anatomic LUCL reconstruction (autograft or allograft) with attention to surgical technique and early protected motion. Early recognition and intervention improve functional recovery and reduce recurrent instability. Contemporary series report good-to-excellent outcomes in approximately 85 to 90% with a high return to activity when rehabilitation is structured. Special considerations are required in deformity-driven cases such as cubitus varus, where corrective osteotomy may be required in addition to ligament reconstruction. Practical pathways for diagnosis and treatment are presented to reduce missed diagnoses and improve outcomes through timely recognition and appropriate intervention. A comprehensive literature search of peer-reviewed articles was performed using PubMed and Medical Literature Analysis and Retrieval System Online (MEDLINE) without date limits, focusing on seminal and contemporary literature and supplemented by manual reference screening.

## Introduction and background

The elbow is intrinsically stable owing to the congruent morphology of the distal humerus, proximal ulna, and proximal radius, together with robust capsuloligamentous restraints and coordinated dynamic muscle forces. Functionally, it behaves as a trochoginglymoid joint, with flexion-extension at the ulnohumeral and radiocapitellar articulations and forearm pronation-supination at the proximal radioulnar joint [1,2]. Stability arises from static and dynamic contributors. The coronoid, olecranon, and radial head provide the primary bony buttresses. The medial and lateral collateral ligament (LCL) complexes and capsule provide soft-tissue restraint. The biceps, brachialis, triceps, and anconeus generate compressive stabilising forces and proprioceptive control [1,2].

Posterolateral rotatory instability (PLRI) is the most common pattern of elbow instability and is classically attributed to insufficiency of the lateral ulnar collateral ligament (LUCL) within the LCL complex [3]. The typical mechanism is a varus-external rotation load applied to a supinated, slightly flexed, axially loaded elbow, which is often due to a fall on the outstretched, supinated hand, producing posterolateral subluxation of the ulna-radial head complex relative to the distal humerus and a pivot-shift phenomenon [3,4]. PLRI may also follow iatrogenic lateral insufficiency after injections or surgery for lateral epicondylitis or after fixation of lateral epicondyle fractures, and it can develop in the setting of cubitus varus, which increases lateral opening and rotatory moments and promotes chronic attenuation of the lateral stabilisers [5,6].

PLRI is frequently under-recognised because symptoms may be vague, standard radiographs are often normal, and instability is dynamic [7,8]. When suspected, a structured approach integrating clinical provocation with targeted imaging reduces missed diagnoses [7]. Contemporary reconstructions of the LUCL generally achieve good to excellent results when followed by structured rehabilitation. Failures are more likely with tunnel malposition or inadequate fixation, or when deformity is unaddressed [9-11]. 

This narrative review synthesises applied anatomy and pathomechanics with clinical presentation and diagnostic strategy. It clarifies when each imaging modality adds value, including the role of dynamic fluoroscopy or ultrasound when static studies are equivocal. It then covers management from non-operative care to operative strategies, summarises outcomes and complications, and explains when deformities such as cubitus varus should be addressed alongside ligament surgery [8-11]. The aim is to reduce missed diagnoses, to place imaging within a clinically coherent pathway, and to align operative decision-making and rehabilitation with the mechanisms that generate instability.

## Review

Methods

This narrative review draws on iterative searches of PubMed and Medical Literature Analysis and Retrieval System Online (MEDLINE) together with manual screening of reference lists from key clinical and biomechanical papers, without date limitation. Searches were updated through October 2025. Sources were selected for relevance and influence on contemporary practice rather than a protocolised systematic review with inclusion and exclusion criteria. The search focused on key clinical questions in PLRI, including mechanism, bedside provocative testing, imaging selection (static and dynamic), non-operative care and bracing, surgical indications and technique, deformity-driven cases such as cubitus varus, and postoperative rehabilitation, and we cited studies that directly addressed those questions. Peer-reviewed clinical, cadaveric, and biomechanical studies, and major reviews, were prioritised. No formal risk of bias assessment was undertaken, in line with the narrative scope.

Applied anatomy and biomechanics

**Figure 1:**
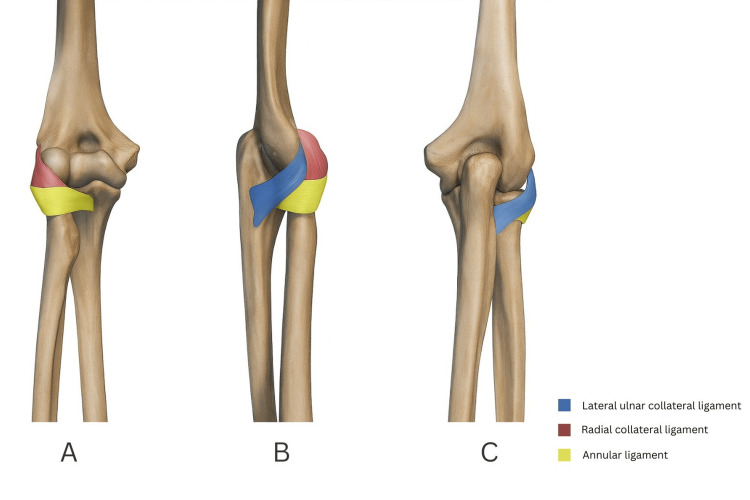
The lateral collateral ligament complex of the elbow Static stabilisers of the lateral elbow, including the radial collateral ligament, annular ligament and lateral ulnar collateral ligament, from different views: (A) anterior view, (B) lateral view, (C) posterior view. This illustration has been created by the authors.

The LCL complex comprises the radial collateral ligament, the annular ligament and the LUCL, as shown in Figure 1 [1,12]. An additional “accessory” lateral collateral band has been described as a variable thickening contiguous with the annular complex rather than a constant, discrete ligament, underscoring anatomical variability [13]. The LUCL extends from the lateral epicondyle to the supinator crest of the ulna, forming the essential posterolateral buttress of the ulnohumeral articulation [12,14]. The LUCL is functionally near-isometric and resists combined varus and external rotation moments, with the humeral origin situated close to the instantaneous centre of rotation of the elbow and the ulnar insertion positioned posterior to the radial notch such that posterolateral translation places the construct under tension [12,14]. Dynamic stability from the triceps, anconeus, biceps, and brachialis increases compressive forces across the radiocapitellar and ulnohumeral joints and augments ligamentous restraint, while capsulomuscular proprioception modulates co-contraction patterns that resist subluxation [2].

When the LUCL fails, the ulna externally rotates and translates posterolaterally beneath the distal humerus, and the radial head subluxes on the capitellum during extension and early flexion, particularly in forearm supination, reproducing the characteristic pivot-shift [3]. Fixed varus malalignment shifts the joint reaction vector and increases lateral opening moments; even modest cubitus varus amplifies posterolateral rotatory torque on the LUCL and predisposes to attenuation and failure over time, explaining tardy PLRI in post-traumatic deformity [6,15].

Epidemiology

A reliable population incidence for PLRI has not been established, and under-recognition likely depresses observed rates [7, 8, 16]. Referral cohorts of patients with lateral elbow pain show that mislabelling at first presentation is not uncommon, often as lateral epicondylitis [7, 8, 16]. In one tertiary referral series of 189 patients, 11% were initially misdiagnosed, and PLRI was the most frequently missed condition, with six of 189 cases (3.2%) [16]. For context, simple elbow dislocation occurs in about five to seven per 100,000 people each year, but most PLRI present without frank dislocation and are therefore not captured by dislocation registries [17].

Clinical presentation and physical examination

Patients commonly describe lateral elbow pain with a sense of giving way, clicking, or a fleeting clunk that is provoked when axial load, valgus stress, and forearm supination coincide, for example, when rising from a chair, doing tabletop or floor push-ups, pushing a door with the elbow extended, or transferring weight through the upper limb [3,18,19]. Symptoms often improve as the elbow moves into greater flexion, and many patients adopt protective strategies that avoid load in extension and supination [3,4]. A history of a fall on the outstretched hand, prior dislocation or subluxation, corticosteroid injection, or previous surgery involving the lateral elbow or long-standing cubitus varus increases pre-test probability and should prompt targeted examination [5, 6]. Vague pain alone is non-specific; it is the combination of pain with mechanical symptoms in the appropriate context that heightens suspicion for PLRI [7, 8].

Inspection may be entirely normal at rest. A subtle posterolateral “dimple” can occasionally be seen in extension, and tenderness is often localised to the lateral epicondyle and the radial head-neck junction rather than the common extensor origin alone [3,4]. Range of motion is usually preserved, though patients may guard against terminal extension. Reproduction of pain or apprehension by axial loading in extension and supination is more discriminating than routine varus-valgus stress testing in neutral [4,19].

**Figure 2:**
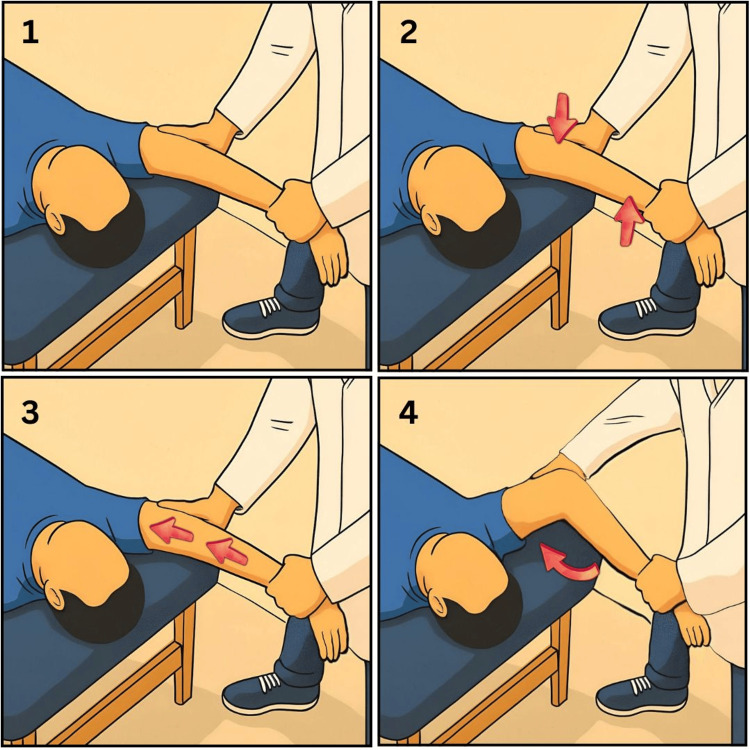
Lateral pivot-shift test of the elbow This illustration demonstrates the combined forces applied during the lateral pivot-shift test as described by O’Driscoll et al. [3]. The sequence is (1) elbow in extension and supination, (2) valgus stress, (3) axial loading, and (4) elbow flexion. This illustration has been created by the authors.

The posterolateral rotatory pivot-shift manoeuvre remains the reference provocative test. With the patient supine, the examiner maintains the forearm in supination, applies a valgus and axial load, and flexes the elbow from full extension as shown in Figure 2; posterolateral subluxation of the radial head on the capitellum may be palpable or visible during early flexion and typically reduces spontaneously beyond about 40° [3]. In awake patients, muscle guarding may limit frank subluxation, so apprehension or pain during the manoeuvre is accepted as a positive finding; under anaesthesia, the test is easier to perform and more specific [3, 4].

Functional tests complement the pivot shift when guarding is prominent. The chair push-up test reproduces symptoms as the patient rises from the chair arms with forearms supinated and elbows near extension; the tabletop test elicits apprehension or a sense of instability during a partial push-up against a desk; and the supine push-up test offers a similar stimulus while controlling trunk load. These are considered positive if they reproduce apprehension, instability, or a subluxation sensation in early flexion [18, 19]. The posterolateral drawer test, performed at roughly 40 to 60° of flexion, may demonstrate posterolateral translation of the proximal ulna-radial head complex relative to the distal humerus compared with the contralateral side [18, 4]. Taken together, a consistent history, a positive apprehension response to at least one provocative task, and lateral-sided tenderness away from the common extensor origin form a persuasive clinical triad for PLRI, even when plain radiographs are normal [7, 8].

Imaging 

A clinically led diagnostic approach avoids over-reliance on normal static imaging and under-appreciation of history-linked apprehension in extension and supination [20]. In practice, the process begins with a symptom pattern and a convincing provocation response. Plain radiographs are obtained first to exclude fracture and malalignment. Cross-sectional imaging is reserved for defined questions, and dynamic imaging is deployed when clinical suspicion remains high despite non-diagnostic static studies [4,7].

Plain radiographs are the first investigation and should include true anteroposterior and lateral views with careful assessment of the radiocapitellar relationship, ulnohumeral congruence, small avulsions from the lateral epicondyle, and subtle signs of malalignment; oblique projections can better profile the radial head-neck junction and the capitellum when suspicion persists. Even when normal, radiographs provide an indispensable baseline and may reveal indirect clues such as a tiny lateral epicondylar fleck or a subtly widened radiocapitellar interval [7,18]. Computed tomography (CT) is most useful when fracture-dislocation is suspected clinically or when plain films suggest complex osseous injury. CT precisely defines coronoid, radial head, and capitellar fractures and facilitates preoperative planning with three-dimensional reconstructions. In patients with recurrent lateral symptoms after an apparently simple dislocation, CT can unmask subtle osteochondral impaction or a malreduced fragment that perpetuates mechanical symptoms and should be addressed before or in concert with ligament reconstruction [21,22].

Magnetic resonance imaging (MRI) directly evaluates the lateral soft-tissue stabilisers. Discontinuity, attenuation, or oedema within the lateral ulnar collateral ligament and adjacent capsule may be demonstrated, and osteochondral injury of the capitellum or radial head is frequently identified. Because reported sensitivity for partial fibre injury is variable, a normal MRI does not exclude dynamic instability when the clinical picture is strong [23,24]. When conventional sequences are equivocal, MR arthrography can increase the conspicuity of partial ligament and capsular defects and improve diagnostic confidence [23,24]. Arthroscopy is not a first-line investigation in suspected PLRI and is used mainly when surgery is planned, where it can confirm instability directly and address intra-articular pathology [25].

Dynamic imaging closes the loop between structural information and functional diagnosis. Fluoroscopic assessment during a controlled pivot-shift manoeuvre can demonstrate posterolateral translation of the radial head relative to the capitellum with spontaneous reduction in early flexion, thereby reproducing the patient’s symptoms in real time. Dynamic ultrasonography in experienced hands can reveal radiocapitellar gapping or loss of continuity of the lateral ligamentous envelope during provocation, with the advantages of being radiation-free and repeatable at the bedside. These modalities are particularly valuable when clinical suspicion remains high despite non-diagnostic radiographs and MRI [20,26].

A pragmatic pathway, therefore, begins with a focused history and a single convincing provocation response, proceeds to radiographs to exclude fracture and malalignment, adds MRI or MR arthrography when lateral soft-tissue injury needs documentation or when operative planning is contemplated, and employs dynamic fluoroscopy or ultrasound when doubt persists. The aim is not to create a test-heavy algorithm but to ensure that imaging is ordered for questions that matter and that dynamic techniques are considered whenever static studies fail to reconcile symptoms with pictures [4,20]. The practical indications, advantages and limitations of imaging modalities are summarised in Table 1.

**Table 1:** Practical indications, advantages and limitations of imaging modalities Created by the authors from the following sources: plain radiographs [12,18], computed tomography (CT) [21,22], magnetic resonance imaging (MRI) [23,24], MR arthrography [23,24], fluoroscopy during provocation [3,20], dynamic ultrasonography [26]. PLRI: posterolateral rotatory instability

Imaging modality		Indication	Advantages	Limitations
Static imaging	Plain radiographs	Plain radiographs are indicated as the first-line assessment in suspected PLRI.	Allow assessment of joint alignment and detection of bony injuries.	They are often normal in isolated PLRI.
	CT scan	CT is indicated when a fracture-dislocation is clinically suspected or when further osseous assessment is required after radiographs suggest complex injury.	It provides precise delineation of fractures and enables three-dimensional surgical planning.	It involves ionising radiation, is prone to metal artefact and offers poor soft-tissue detail.
	MRI	MRI is indicated for suspected soft-tissue injury.	It characterises ligamentous and capsular injury patterns and detects associated osteochondral lesions.	It does not exclude dynamic instability and has variable sensitivity for partial tears.
Dynamic imaging	MR arthrography	MR arthrography is indicated when conventional MRI is equivocal, yet clinical suspicion remains high, or when partial-thickness ligament or capsular tears are suspected.	It improves detection of small tears, localises and defines the extent of injury and can reveal capsular leakage or small intra-articular pathology.	It is invasive, carries contrast-related risks and is technique-dependent.
	Fluoroscopy	Fluoroscopy is indicated when static imaging is equivocal in the context of high clinical suspicion or when symptoms are position-dependent.	It offers real-time dynamic visualisation of alignment during stress manoeuvres and has high sensitivity for positional subluxation.	It is technique-dependent and may be non-diagnostic unless the manoeuvre reproduces the patient’s symptoms.
	Ultrasound	Ultrasound is indicated when static imaging is non-diagnostic despite high clinical suspicion or when symptoms are position-dependent.	It provides real-time dynamic assessment at the bedside and is radiation-free and repeatable.	It is operator dependent and may be non-diagnostic unless the provocation reproduces the patient’s symptoms.

Management 

Management is determined by symptom burden, demonstrable mechanical instability, chronicity, functional demands and contributory anatomy (e.g., cubitus varus). A short, structured trial of non-operative care is reasonable when instability is low-grade or equivocal, symptoms are improving after a recent injury, or the patient prefers to avoid surgery. Sustained mechanical apprehension, reproducible giving way on provocative testing, recurrent instability or failure of a supervised non-operative programme are indications to proceed to operative stabilisation. Acute avulsions with a reparable pattern may be suitable for primary repair, whereas chronic insufficiency more often requires an anatomic LUCL reconstruction [19,27,28].

Non-operative management

Defined as activity modification, targeted physiotherapy and time-limited bracing with education on provocative positions [27,29]. A defined four- to six-week trial is typical when symptoms are improving and mechanical apprehension is low-grade [19,27,29]. Non-operative care focuses on unloading the injured lateral complex while restoring dynamic stability. Activity is modified to avoid axial loading in extension and supination, and physiotherapy emphasises the extensor-supinator mass, coordinated co-contraction of biceps and triceps to generate compressive stability across the ulnohumeral and radiocapitellar joints, scapular control, and proprioceptive retraining [19,27,29]. Short courses of analgesia can be used judiciously to facilitate early motion without provoking varus or supination torques [19]. This approach is most appropriate for patients with mild, improving symptoms and no clear mechanical reproduction on examination [19,27]. Chronic mechanical PLRI rarely resolves with rehabilitation alone once the LUCL is incompetent; persistent apprehension or instability after a defined course should trigger surgical planning [19,27,29].

Bracing

A hinged brace worn with the forearm in pronation reduces varus moments and limits supination during the vulnerable early phase [19]. In acute soft-tissue injury at risk of instability, four to six weeks of protected motion with an extension block is typical, followed by supervised weaning and progressive range of motion [19,27]. Bracing can be used as an adjunct during a non-operative trial while dynamic control is rebuilt. The brace is unlocked in stages as pain settles and stability improves, with strict avoidance of varus loading throughout early rehabilitation [19,27].

Surgical management 

Indications and Planning

Surgery is considered when pain, apprehension, or giving way persists after structured conservative care, when provocative testing is convincingly positive, when imaging corroborates lateral soft-tissue insufficiency, or when instability limits work or sport [28]. This includes cases of iatrogenic lateral insufficiency after lateral epicondylitis procedures when symptomatic instability is present [6]. Acute avulsion from the lateral epicondyle with good-quality tissue and a discrete footprint can be repaired early, whereas subacute or chronic cases with attenuation or poor tissue quality are better treated with anatomic LUCL reconstruction using a tendon graft [28]. Planning confirms the clinical pattern, reviews the lateral epicondyle and the supinator crest on imaging, and screens for varus malalignment that increases posterolateral torque. Corrective distal humeral osteotomy is added when varus alignment is material because isolated ligament reconstruction in this setting risks early failure [6,28]. Concomitant pathology like osteochondral injury, loose bodies, and stiffness is identified and addressed in the same sitting where appropriate [12,30].

Technique and Intraoperative Checks

A lateral approach through the Kocher interval between the anconeus and extensor carpi ulnaris provides reliable access to the lateral epicondyle, radiocapitellar joint, and supinator crest [12,14,19]. A Kaplan interval is selected when broader joint exposure is required [12,14]. The posterior interosseous nerve is protected by maintaining the forearm in pronation during deep dissection and avoiding forceful retraction over the supinator [12,19]. Graft selection is individualised. Palmaris longus autograft offers ease of harvest with minimal donor morbidity, gracilis provides predictable length and diameter when a larger graft is desirable, and a partial triceps slip avoids a second harvest site and supplies robust local tissue. Allograft is reasonable when donor morbidity is a concern [10-12,28]. Fixation, whether interference screws, cortical buttons, or suture anchors, should be secure and compatible with local bone stock to tolerate early protected motion [12]. The aim is robust fixation without compromising isometry across the arc of motion [12,14].

Accurate tunnel position and control of length change dominate success. The humeral origin is placed just posterior and inferior to the lateral epicondyle near the instantaneous centre of rotation [12]. The ulnar insertion targets the crista supinatoris to create a posterior sling that tensions with posterolateral translation [12,14]. A passing suture or trial tape along the intended path is cycled from extension to approximately 90° in pronation; minimal length change across the arc indicates acceptable isometry [12]. Isometry is confirmed intraoperatively across the arc of motion and adjusted as required. Care is taken to avoid tunnel convergence with prior hardware or with planned osteotomy cuts [12]. Graft passage begins on the ulna to establish the sling, then fixation on the humerus follows with the forearm in pronation and the elbow flexed about 30 to 45° while a gentle valgus load is applied [12]. The elbow is cycled through flexion and extension in pronation to confirm stable reduction without overtightening that would limit motion, and full forearm rotation is checked at approximately 90° of elbow flexion to exclude impingement or graft abrasion [12].

Intraoperative assessment is pragmatic. A controlled pivot shift under fluoroscopy, where available, is used to confirm that posterolateral translation has been eliminated and that reduction is maintained through early flexion [20]. The radiocapitellar joint is inspected during provocation to ensure congruence, and loose bodies or focal chondral lesions are addressed to avoid mechanical symptoms that could confound recovery [12]. In early avulsion patterns from the lateral epicondyle with adequate tissue quality, primary repair using suture anchors or transosseous sutures can restore stability; limited augmentation with suture tape may be considered for borderline tissue [19,28]. Repair is reserved for early cases with a clear footprint and without fixed deformity or chronic attenuation [19,28]. Arthroscopic or arthroscopic-assisted reconstruction permits precise anchor placement and guided graft passage with excellent visualisation during provocation; early series report outcomes approaching those of open techniques in selected patients, although most high-grade chronic cases are treated open [25,31-34]. Selection should reflect the surgeon's experience and equipment availability [25].

Outcomes, Pitfalls, and Revision

Anatomic reconstruction restores stability in most symptomatic elbows when tunnels are accurate and rehabilitation is structured and systematic, and contemporary series consistently report restoration of stability in roughly nine in 10 elbows, good to excellent patient-reported outcomes in most cases, and low revision rates after primary reconstruction [9-11,35]. Mid-term triceps autograft cohorts report mean Mayo Elbow Performance Scores around 90 with very low recurrence [10,11]. Prognosis worsens when varus deformity is uncorrected, when tunnels are malpositioned or non-isometric, or when early rehabilitation permits varus loading [6,12,14,15,30]. Complications are mitigated by meticulous technique and disciplined rehabilitation: stiffness is limited by accurate tunnel placement, secure fixation, and early protected motion [12,29,30]; posterior interosseous nerve irritation is minimised by maintaining forearm pronation during deep dissection and using gentle retraction [12]. Surgeons should keep a high index of suspicion for heterotopic ossification when pain and loss of motion are disproportionate, as periarticular heterotopic bone can arise at atypical soft-tissue locations; careful soft-tissue handling and avoidance of excessive periosteal stripping are prudent [36]. Recurrent instability most often reflects tunnel malposition, non-isometric reconstruction, or humeral fixation failure; prevention rests on deliberate isometry checks, reliable fixation, and strict avoidance of varus and forced supination during early rehabilitation [12,28,35,37].

When failure occurs, mechanisms are most commonly humeral fixation failure, graft loosening and tunnel malposition. Revision focuses on restoring isometry (with a new humeral start point if needed), providing robust fixation and avoiding tunnel convergence; triceps or hamstring autografts and allografts are all effective options [12,37]. Stability is usually recovered, yet functional gains are typically smaller than after primary reconstruction, and recurrence is more frequent, which should be discussed preoperatively with patients [37].

Surgical Challenges in the Presence of Cubitus Varus

Cubitus varus deformity alters elbow biomechanics, increasing lateral opening and external rotation moments and placing additional varus and rotational stress on the lateral ulnar collateral ligament, which complicates reconstruction and increases the risk of failure [6,15]. When deformity is material to the mechanism, a combined approach with corrective distal humeral osteotomy and LUCL reconstruction is preferred, as isolated ligament reconstruction may fail [6,38]. Recentring the mechanical axis reduces pathological moments and is associated with restoration of alignment and stability with meaningful functional gains [6,38]. Preoperative planning should define the degree of varus and any concomitant ligamentous insufficiency to guide the surgical approach, tunnel trajectories, and fixation strategy, and postoperative rehabilitation must balance early range of motion with protection of both the osteotomy and the reconstruction [6,38].

Postoperative Rehabilitation

Postoperative rehabilitation is crucial to optimise outcomes and restore elbow function. Initial immobilisation is typically seven to 14 days with the elbow at approximately 90° of flexion and the forearm in pronation [27,29]. This is then replaced with a hinged brace, often with an approximately 30° extension block, to maintain pronation and permit protected motion [12,27,29]. During the first six weeks, patients perform active-assisted flexion and extension in pronation and carry out forearm rotation at approximately 90° of elbow flexion [12,19,29]. At about six weeks, the brace is progressively unlocked with progression to motion in neutral and then in supination [12,29]. Strengthening usually begins once a comfortable range is established, around eight weeks, focusing on the extensor-supinator mass and coordinated biceps-triceps co-contraction [11,19]. The brace is generally removed between eight and 12 weeks [11,29,30]. Return to unrestricted activity is permitted after roughly four to six months, with longer timelines for collision or contact demands [11,28]. Throughout all phases, varus loading and forced supination should be avoided [12,27,29], and clinicians should remain vigilant for stiffness and heterotopic ossification when pain and loss of motion are disproportionate [36]. Early, structured rehabilitation within these parameters has been associated with excellent functional outcomes and high rates of return to sport and daily activity [10,11,28].

Future directions

Despite advances, important gaps remain. Apparent prevalence is likely depressed by under-recognition and initial mislabelling as lateral epicondylitis; prospective epidemiology should define true frequency, risk factors, and natural history [8,16]. Diagnostic research should validate dynamic imaging protocols that can be deployed beyond specialist centres and clarify the incremental value of fluoroscopy and ultrasound when MRI is inconclusive [20,26]. Comparative trials evaluating open versus arthroscopic or arthroscopic-assisted reconstruction, humeral fixation strategies, graft choices, and augmentation (“internal brace”) techniques would better inform operative selection [31-34]. For deformity-driven cases, studies should define indications, timing, and long-term outcomes for staged versus combined distal humeral osteotomy with LUCL reconstruction [6,38]. Rehabilitation science should move beyond expert consensus to compare accelerated and conventional protocols using agreed outcome sets that include patient-reported function and return-to-work metrics [11].

## Conclusions

PLRI is an under-recognised cause of lateral elbow pain and functional apprehension. A high index of suspicion, careful history and examination with targeted provocative testing, and judicious use of static and dynamic imaging help identify dynamic instability that plain films may miss. Non-operative measures have a role in selected low-grade or early cases. Symptomatic or chronic instability is best addressed with anatomic reconstruction of the LUCL, performed with precise tunnel placement and followed by structured rehabilitation. In deformity-driven cases, correction of alignment alongside ligament reconstruction reduces recurrence. Attention to these principles shortens time to diagnosis, improves patient-centred outcomes, and reduces failure through mechanism-based care.
